# Agency Leaders' Assessments of Feasibility and Desirability of Implementation of Evidence-Based Practices in Youth-Serving Organizations Using the Stages of Implementation Completion

**DOI:** 10.3389/fpubh.2018.00161

**Published:** 2018-05-29

**Authors:** Lawrence A. Palinkas, Mark Campbell, Lisa Saldana

**Affiliations:** ^1^Department of Children, Youth and Families, Suzanne Dworak-Peck School of Social Work, University of Southern California, Los Angeles, CA, United States; ^2^Oregon Social Learning Center, Eugene, OR, United States

**Keywords:** innovation, adoption, feasibility, desirability, evidence-based treatments and practices, qualitative methods, youth mental health, SIC

## Abstract

**Background:** This study examined influences on the decisions of administrators of youth-serving organizations to initiate and proceed with implementation of an evidence-based practice (EBP).

**Methods:** Semi-structured interviews, developed using the Stages of Implementation Completion (SIC) as a framework, were conducted with 19 agency chief executive officers and program directors of 15 organizations serving children and adolescents.

**Results:** Agency leaders' self-assessments of implementation feasibility and desirability prior to implementation (Pre-implementation) were influenced by intervention affordability, feasibility, requirements, validity, reliability, relevance, cost savings, positive outcomes, and adequacy of information; availability of funding, support from sources external to the agency, and adequacy of technical assistance; and staff availability and attitudes toward innovation in general and EBPs in particular, organizational capacity, fit between the EBP and agency mission and capacity, prior experience with implementation, experience with seeking evidence, and developing consensus. Assessments during the Implementation phase included intervention flexibility and requirements; availability of funding, adequacy of training and technical assistance, and getting sufficient and appropriate referrals; and staffing and implementing with fidelity. Assessments during the Sustainment phase included intervention costs and benefits; availability of funding, support from sources outside of the agency, and need for the EBP; and the fit between the EBP and the agency mission.

**Discussion:** The results point to opportunities for using agency leader models to develop strategies to facilitate implementation of evidence-based and innovative practices for children and adolescents. The SIC provides a standardized framework for guiding agency leader self-assessments of implementation.

## Introduction

In the past two decades, there has been an increased effort to implement evidence-based practices (EBPs) into real world community public social service settings (1, 2). To do so successfully, agency leaders are necessarily involved in extensive planning and self-assessments of the organization's capacity to engage in training and quality assurance in the EBP, which often involves a complex set of interactions among developers and system leaders, front line staff, and consumers (3). In fact, it is generally understood that it takes an agency a minimum of 2 years to complete the full implementation process (4) for psychosocial interventions and that the success of a program is strongly influenced by the presence or absence of certain barriers and facilitators to implementation (5–8) as well as the strategies selected to facilitate the implementation (9–11). Yet, there remains much to be learned regarding which aspects of these methods and interactions are most valuable for successful installation of new practices (8) and which are considered by agency leaders when conducting self-assessments throughout the full implementation process.

There is consensus that implementation of psychosocial interventions within social service settings is a recursive process with well-defined stages or steps (5, 12). Fixsen and Blasé (4) described several stages that are not necessarily linear and that impact each other in complex ways. In the case of EBPs, an intervention developer, technical assistance purveyor, or third-party intermediary typically assists programs in navigating their way through the implementation process to ensure that the program elements are delivered in the manner intended by the developers. Nevertheless, there remains a dearth of methods to accurately assess the key processes involved in the implementation stages and the fidelity of the implementation methods assessed (13, 14).

Given the non-linear, yet staged progression of the implementation process, the measurement of this process must be flexible enough to capture these potential variations. Having a well-defined measure for assessing implementation to inform knowledge about the typical progression through the stages of implementation helps to increase the likelihood that sites can use information garnered in the early stages to inform and support their success in later stages (5, 8). There might be particular value in giving agencies feedback regarding their progress during the early implementation stages to help them assess and potentially calibrate their efforts to proceed, or to reassess whether their current implementation plan remains viable (3, 15). The Stages of Implementation Completion [SIC; (3)] was developed to meet this need.

### The stages of implementation completion (SIC)

The SIC is an 8-stage assessment tool (12) developed as part of a large-scale randomized implementation trial that contrasted two methods of implementing Treatment Foster Care Oregon [TFCO (formerly Multidimensional Treatment Foster Care); 16)], an EBP for youth with serious behavioral problems in the congregate care and child welfare systems. The eight stages range from Engagement (Stage 1) with the developers/purveyors in the implementation process, to achievement of Competency in program delivery (Stage 8). The SIC was developed to measure a community or organization's progress and milestones toward successful implementation of the TFCO model regardless of the implementation strategy utilized. Within each of the eight stages, sub activities are operationalized and completion of activities are monitored, along with the length of time taken to complete these activities.

As described in Methods, the current paper is part of a larger trial focused on adapting the SIC for additional EBPs within youth-serving systems, and assessing its utility in measuring implementation success across service sectors including schools, substance abuse treatment, and juvenile justice (3). Sub activities within each stage target specific tasks necessary to complete each stage within the particular EBP. For example, Readiness Planning (Stage 3) is required for the implementation of all EBPs, but one TFCO specific sub activity is to conduct a foster parent recruitment review. The SIC is date driven in order to analyze the pace of implementation, and to identify when agencies experience hurdles in their process that might delay success. The SIC also yields a proportion score which takes into account the number of activities within a stage that are completed. Thus, scores for both the speed and the proportion of activities are calculated to determine if such factors influence the successful adoption of an EBP. The SIC maps onto three phases of implementation including Pre-Implementation, Implementation, and Sustainment.

### Agency leader considerations for implementation

Although there are numerous frameworks and models for implementing healthcare innovations, each consistently point to several factors believed to be associated with successful adoption, implementation, and sustainment, including characteristics of the inner and outer setting, the intervention itself (i.e., characteristics of the organization and staff involved in the implementation and the external environmental factors that determine the demand for the intervention and the resources available for its implementation, respectively, and the population served by the practice (5–8). As Proctor et al. (17), p. 72) have observed, “the success of efforts to implement evidence-based treatment may rest on their congruence with the preferences and priorities of those who shape, deliver, and participate in care.” Similar pragmatic arguments have been made by Bhattacharyya et al. (18) who pose that theory is not necessarily better than “common sense” for guiding implementation. For example, highlighting the importance of fiscal viability and feasibility, a study by Palinkas et al. (19) found that directors of youth-serving mental health clinics made decisions to adopt or not adopt innovative EBPs based on an assessment of costs and benefits associated with adoption, capacity for adoption, and acceptability of new practices. Moreover, assessment of costs and benefits exhibited several principles of behavioral economics including loss aversion, temporal discounting, use of heuristics, sensitivity to monetary incentives, decision fatigue, framing, and environmental influences. However, the extent to which these preferences and priorities change from one stage of implementation to the next (19) and how decision-makers self-assess the weight of these considerations remains unknown.

### Current paper

The current paper aims to disentangle these targets by integrating firsthand knowledge from agency leaders working within youth-serving systems when attempting to implement one of three EBPs for the treatment of serious emotional problems in youth. To better understand the key factors involved in deciding whether or not to adopt, support, and sustain one of these practices, we conducted a qualitative study focused on the assessment of feasibility and desirability of the EBP, as well as the efforts made for implementation by mental health and social service agencies representing different youth-serving systems. Our aim was to examine the factors that influenced these self-assessments made by agency leaders at each stage of implementation, as measured by the SIC.

## Methods

### The SIC study

The study described in this paper was part of a larger effort to determine if the SIC can be applied across EBPs from different service sectors and accurately predict successful implementation outcomes. Naturally occurring implementation attempts were observed toward program start-up and sustainment of three widely-used EBPs that operate in various contexts and child public service sectors: Multisystemic Therapy [MST; (20)], Multidimensional Family Therapy [MDFT; (21)], and TFCO (16). Selection criteria for inclusion in this study included being an EBP: (a) for child and family mental health delivered within key service sectors (schools, juvenile justice, substance use, child welfare); (b) large real-world uptake within the respective service sectors in order to achieve sample size requirements for study analyses; and (c) EBP developers who expressed interest in using the SIC and in advancing understanding of the universal implementation elements shown to increase the successful uptake of EBPs.

### Participants

A random sample of 5 sites, from each of the three participating EBPs, that had advanced minimally through Stage 3 (Readiness Planning) or beyond were recruited for participation. Identified sites were asked to consent to participate in a one-time semi-structured qualitative interview conducted by phone. Participants were compensated ($45) for their time. Consenting procedures included a request for use of the sites' SIC data being collected as part of the larger study. Of the 15 sites identified, 14 agreed to participate in this phase of the overall study.

A total of 19 individuals participated across the 14 agencies, with three agencies including two representatives, and one agency including three representatives. Agencies were asked to purposefully select individuals within their agency that were responsible for decision making regarding the program, and who were involved in the implementation planning. The majority of the participants (68.4%) were female, and ages ranged from 31 to 63 years. Participants included agency leaders (i.e., chief executive officers [CEO], executive directors, or deputy directors; 31.6%); program specific directors (42.1%); or clinical supervisors (26.3%). The study was approved by the Institutional Review Board at the Oregon Social Learning Center.

### Data collection

Semi-structured interviews were conducted over the telephone by two project investigators, either in tandem or separately, using a standardized interview guide organized using the SIC as a framework. Questions focused on behaviors and perceptions regarding implementation activities identified on the SIC (e.g., “What steps or processes did you go through at your agency before getting started to determine if the EBP would be a good fit for your agency?”). Information collected during the interviews included characteristics of person being interviewed, role in agency, involvement in implementing the EBP, the process of implementation at each of the SIC stages that were completed by the agency, potential for sustainment of the EBP, reflections on consistency of the EBP with participant and agency goals, perceived costs and benefits associated with implementing the EBP, primary changes in any of the existing policies and procedures that were necessary to implement the EBP, and anything in particular the participant or agency staff did or did not like about the EBP in general. Interviews lasted approximately 1 h and were audio recorded, with written notes taken and used to supplement recordings when the audio quality was poor.

### Data analysis

Interviews were transcribed verbatim. Transcripts were reviewed for accuracy by two members of the research team. Deductive and inductive thematic coding was employed in this analysis (22). Deductive codes were based on interview questions, and inductive codes were based on responses by agency leaders. Data were coded according to Saldaña (23) first- and second-cycle coding method. During the first coding cycle, three researchers independently reviewed and open-coded these raw materials. They then undertook “consensus coding” together, a process used to establish agreement and increase the rigor and validity of coding in qualitative research (24, 25). Lists of codes developed individually by each investigator were subsequently discussed, matched and integrated into a single codebook. Inter-rater reliability in the assignment of specific codes to specific transcript segments was assessed for a randomly selected transcript. For all coded text statements, the coders agreed on the codes 81% of the time, indicating good reliability in qualitative research (26). A web-based qualitative data management program (27) was used for coding and generating a series of categories arranged in a treelike structure connecting text segments as separate categories of codes or “nodes.” Through repeated comparisons of these categories with one another, these nodes and trees were used to create a taxonomy of themes that included both *a priori* and emergent categories and new, previously unrecognized categories. The SIC was used as a framework for organizing the first order codes, while grounded theory analytic methods (28) were used to construct themes based on the inductive codes.

## Results

Analysis of the self-assessments made by system leaders in the course of adopting, supporting and sustaining the three EBPs revealed distinct influences on the decisions about the feasibility and desirability of the EBP and its implementation as measured by each of the three SIC phases—Pre-Implementation, Implementation, and Sustainment (Table 1). Each of these sets is described below with an emphasis on themes related to the intervention, outer (external factors such as policy and funding) and inner (internal factors such as staffing and attitudes) settings, and the interaction of these factors.

**Table 1:** Influences on agency leadership assessment of feasibility and desirability.

**Pre-implementation**	**Implementation**	**Sustainment**
**INTERVENTION**		
Affordable		
Flexible	Flexible	
Requirements	Requirements	
Validity, reliability and relevance		
Cost savings		Costs and benefits
Positive outcomes		
Adequacy of information		
**OUTER SETTING**		
Funding for EBP	Funding for EBP	Funding for EBP
Support from sources external to agency		Support from sources external to agency
Adequacy of technical assistance	Adequacy of training and technical assistance	
Demand for EBP		Need for EBP
Relations with outsiders	Getting referrals	
**INNER SETTING**		
Staff availability	Existing or new staff	
Staff attitudes		
Organizational capacity	Implementing with fidelity	
Fit between EBP and org mission		Fit between EBP and mission
Fit between EBP and org capacity		
Prior experience with implementation		
Experience seeking information		
Developing consensus		
Relations with insiders		

### Pre-implementation phase (SIC stages 1–3)

Preliminary assessments of feasibility and desirability of the EBP and its implementation at the Pre-Implementation phase corresponded to characteristics of the intervention, the outer and inner settings common to many implementation frameworks (5–7).

#### Intervention

Characteristics of the intervention that system leaders considered included its affordability, flexibility, requirements, validity, reliability, relevance, evidence of positive outcomes, and potential cost savings. For instance, one EBP was selected by an agency because “it was also a bit more affordable” and because “[EBP] is a much more flexible type of therapy to implement. And by flexible, just, I mean that in every sense of the word” (program director). On the other hand, one of the agencies that eventually elected not to proceed with a different EBP, instead selected an intervention that they judged to be less intrusive and burdensome. This same EBP was indeed selected by a different agency though due to its rigor as stated by one agency CEO, “Well basically, we, you know, are juvenile justice and we wanted a program that was valid, and reliable, and tested on an urban juvenile justice population with the challenges that our kids and families face.”

Agency directors also evaluated EBPs during the Pre-Implementation phase for their potential to produce cost savings for the agency. As described by one director, “Well, feasibility goes hand in hand with money…that's what motivates everyone when you're in a non-profit is that, you know, if I have an investment in staff, which is money, and I want a cost saving as it relates to [saving] kids from escalating, then it's about how can I get the best bang for my buck?” Cost savings, in turn, are related to outcomes: “So we needed something that was intensive but over a long enough duration, to get us the outcome that we wanted… and then we looked at cost savings, in so far as, you know, we looked at that we wanted to keep kids in their community vs. having kids go out of home to a residential placement” (CEO).

#### Outer setting

Characteristics of the outer setting included availability of funding and support for implementation, external demand for the intervention, and interorganizational relationships. Availability of funding was the most frequently cited characteristic of the outer setting during the Pre-Implementation phase. As one agency director noted, “We were definitely sure that we thought it would be a good fit with our background in treatment foster care. The main question was just whether or not we could get some funding to start it up.”

A second characteristic of the outer setting was the availability of technical assistance from the treatment developers. As noted by one CEO, “I guess the thought process that helped, that really aided in deciding that we would implement [EBP] was all of the support … they were able to review sample policies, give me feedback on those things, before we ever needed to kind of hit the ground running…I would say that's a large pro, and that is probably what helped us [to] decide, okay, let's do this. Because, otherwise, we may not have been so quick to move into implementation” (CEO).

A third characteristic of the outer setting was an external demand for the intervention. Agencies that worked with larger service systems such as child welfare and juvenile justice would often take into consideration the goals, preferences, and mandates imposed by these systems. For instance, “…the goal of Probation, uhm, then and now is they really were looking for something that was, uh, working with families, working with these clients, and really preventing residential care. So, they knew it needed to be something, you know, intensive, something that really looked at, you know, multiple systems with the ultimate goal of keeping kids out of residential care or out of detention… so they were really looking at, ‘Can these models really provide that kind of outcome that we're looking for?”’ (supervisor).

Finally, agency assessment of feasibility and desirability were based on the nature of social interactions with external organizations and representatives, including potential collaborators and sources of financial support. For example, one program director noted, “So we met with them as well as with our mental health board and throughout our community just to be sure as we're going into this process that we weren't necessarily wasting our money and time that no one was going to use this service” Whereas other valuable interactions included intermediaries that provided assistance with program development. For example, “Sometimes, we needed kind of an objective person who wasn't necessarily part of any of us to bring us together, more of, to appear to bring in the program. So definitely helping with the stakeholders, I guess, would be what I'm trying to say. So they assisted with stakeholder meetings. They also were very instrumental in helping us put together our risk assessment tools.”

#### Inner setting

During Pre-Implementation, characteristics of the inner setting that agency leaders included in their self-assessment included staffing requirements, staff attitudes toward the EBP, organizational capacity, fit between the EBP and agency mission and organizational capacity, prior experience with implementing EBPs, experience seeking information about EBPs, and building consensus. Considerations of staffing patterns focused on whether current staff were available to implement the EBP or additional staff needed to be hired. In some instances, agencies felt they could implement with current staff, as noted by one supervisor, “We already had staff. You know what I mean? We, uhm, didn't have to do a bunch of recruiting people or anything like that.” In other instances, agencies perceived a need to hire additional staff and had to consider the feasibility of successfully doing so, “We looked at, uhm, the level of staff that were required, and would we be able to tap into that level of staff here in our area or geographic area? Would we have to be looking outside of that? Would it even be possible to, uh—to get those—that level of staff on board?” (program director).

Directors also considered whether existing staff would be in support of changing their practice and adopting new interventions. In one agency that was not successful in its efforts to implement [EBP], a clinical supervisor explained: “So what happened was some of our team members were not quite ready. They were not as open to the ideas of new evidence-based practice, which is not working and a change and being different with clients than normal, you know?” In another agency, the clinical director stated: “I don't think there were any major surprises, not to me any major surprises, but there were some philosophical ideas that [EBP] really focused on that were a stretch for some of the staff and administration.”

Similarly, the capacity of the agency to implement something new also was assessed in terms of availability of resources for training and staff retention. As noted by one executive director, “In those days we didn't do a whole bunch of high-tech analysis. Basically it was coming from direct experience from successes and challenges that we were faced with by just running foster care, being a foster family agency. We knew we had a problem retaining our foster parents and training and supporting them…to be able to deal with the high-end youth challenges. We also knew we needed resources in that department in order to do the work better, and right.”

Another inner setting influence on assessment of feasibility during Pre-Implementation was the nature of social interactions within the agency. For the most part, the interactions were characterized by consensus built on shared values. According to one supervisor, “We did not have to do a lot of internal negotiation before we landed on [EBP]. I provided my clinical director with some of the other options and as we talked about it and reviewed them. We definitely came to believe this fit our agency much better.” An associate executive director of another agency stated: “We didn't know enough to not be on the same page. As it came up, we were like ‘This is amazing. This is amazing. We have to have it to improve our quality of services and our outcomes.’ It was as easy as that.” Yet, in other instances, some level of negotiation was required before consensus was achieved. As described by one program director, “Well, we had to do some negotiations. For one, it's a higher per diem than what they're accustomed to. So some of that was about what all it entailed and helping them to truly see the difference between following that model in comparison to our current foster care, where it's more of an as-needed basis.”

#### Interactions across settings

Importantly, responses by agency leaders highlighted that many of their identified influences transcended more than one setting. For instance, the degree of fit between EBPs and the agency's mission reflects an interaction between characteristics of the intervention with the outer and inner settings. As explained by one agency program director, “We basically researched a few of them, and then, we kind of—I, myself and my executive director met and we tried to figure out which one would be most feasible for our population, our organization, what would fit in with our procedures, um, and was also enhancing our treatment and so on.” In another example, the program director of one agency reported working with the treatment developer to determine how to fit the requirements of the EBP with the capacity of the organization and delivery of the intervention in rural settings, “And then what happened was we would usually discuss that, you know; because there were a lot of requirements that we didn't, we needed to make, to make our system fit those. And they didn't always fit those. So we spent a lot of time just simply on the requirements of [EBP] and how could we make that work in these rural areas.”

A second illustration of the interaction between different influences across settings was the availability of information about the EBP and its implementation. Participants reflected a range of responses to the question of whether they had sufficient information about the EBP and its implementation to make an informed decision. In some instances, agency directors were satisfied with the information they received about the EBP and how to implement it. As stated by one program director, “[EBP] is incredibly thorough. And from the beginning, even before we had contracted with them, they sent us all of the materials and all of the information that we were gonna need. And then throughout the entire process, having been assigned [EBP purveyor] from the agency, we were on the phone with him very regularly, and as well as he was available to us really whenever questions arose… then they assigned an expert to us who remains with us now. Uhm, so we certainly never were at a lack for, ‘How does this work?’ We were given so much information, and the representatives from [EBP] were available to us at any time that we needed them.” Yet other supervisors noted, “having a little bit more information would have made a smoother transition.” Lack of information led to feelings of mistrust and lack of transparency and an underestimation of the time and effort required for successful implementation, “So, we were told it was gonna to be very simple, we could pull them out of treatment, we could meet with the families; we had no idea, um, that, or I had no idea that the trainer was going to be, like, a huge stickler for whatever it takes, go to the home. Like, we were not an agency that was going into the home. I was told we wouldn't really have to go into the home. That very quickly changed… So, we were told the information but it wasn't, I felt like it wasn't completely transparent.”

Related to the availability of information was the agency's experience seeking information. Many participants, for instance, reported conducting their own literature and internet searches for information about the proposed EBP. “I Googled and did research on [EBP], and then I did research, and then I did a Google search on evaluations of [EBP], downloaded those articles, read them, then I'd looked at, you know, how they have an evidence-based practice website. Looked at it then to—based on my population, what would be the best one to go to. [EBP] kept popping up, and so after a while, it kept coming up again and again and again, and then I said, ‘Okay cool.”’ (CEO). Whereas, other participants reported getting advice from others, including treatment developers and usually found this information to be quite helpful in facilitating the decision of whether or not to proceed with implementing the EBP. “After we actually approached [EBP], and we had a series of conversations with him and they would do this like readiness to implement after conversations. When he started telling us all the do's and don'ts, that is when we started saying ‘Whoa, whoa, may not be a good fit after all”’ (CEO). Finally, directors sought information from agencies and other agencies that had implemented or were currently implementing the EBP. “We went to an organization…. for an agency that was already up and running doing [EBP]…so we went there and met with them. I personally met with their staff and their recruiter and it was a 2-day trip for that alone as well as then meetings we had with the mental health board locally and other community providers to present this as an option just to see if there was even buy-in.”

### Implementation phase (SIC stages 4–7)

Similar to Pre-Implementation, self-assessments of feasibility and desirability of the EBP and its implementation at the Implementation phase corresponded to characteristics of the intervention, the outer setting, and the inner setting.

#### Intervention

Influences associated with the intervention itself included its flexibility and requirements for delivering the service. In some instances, perceived flexibility of the EBP requirements made the process relatively easy. As noted by one program director, “The staff that they had were ready, and that is the great part about [EBP]. What I love is that they can do it half-time or full-time, and then you can start out half-time, and work toward getting a full-time [EBP] therapist, which is what we're currently doing.” In other instances, EBP requirements created challenges. The on-call requirement for [EBP] was especially challenging, either because of the lack of available staff or the lack of experience of staff being on call. One program director explained in contracting out to a private agency to deliver [EBP], “I went with a private agency that we had worked with for many years but they have been known to be very effective with substance use in this area, because they have the adult drug court contract; they have been doing it for 25 years. But in that, when you have a private setting like that, they have earned the right at that level to not be on-call all the time. They have also earned the right at that level to not be in-home, if they don't want to. I do think that in-home would have, at least initially, would have been a better fit if we could have figured out a way in this area to do that. The on-call portion has definitely been a huge challenge.”

#### Outer setting

Outer setting influences during the Implementation phase included the availability of funding to deliver the service, availability and adequacy of technical assistance, and availability of appropriate referrals. The limited availability of funding to provide the service to particular clients was cited by some agency directors as an implementation barrier at this phase. For instance, “Uh, the main referral problem we had would be we really serve Medicaid—kids with Medicaid and once in a while—a lot of the kids through Juvenile Justice have Medicaid, uhm, but once in a while there is a—a child that we just are not able to serve because of the Medicaid issue.”

A second important outer setting influence was the availability and adequacy of technical assistance. Most agencies appeared satisfied with the technical assistance received after the initial clinical training, noting high accessibility to an expert EBP consultant with frequent interactions. For instance, one agency director stated that “…compared to how we're usually trained, absolutely; I think this was phenomenal training. And I think that it's as good as it can be. You always think there are things you could do, follow-up training you know, 90, maybe 90 days after you've actually implemented …that's what the EBP experts are there for.” Another director stated: “it was a really steep learning curve. Uhm, and I think it was manageable because we did have weekly consultations. I think that's the only thing that made it doable…” In fact, one program director of another agency stated a desire for even more face-to-face consultation after the initial training: “I mean, the only thing that we would've liked to be different, but we couldn't because of the location of where the trainer was, would be [that] we'd like more face-to-face contact.” Similarly, another director commented, “and one thing about the [EBP] training team…that came out and trained all of us and our other staff, they have always been incredibly helpful to us. They have made it absolutely wonderful and a really easy way to learn. So even though it gets really intense, they're always terrific with us.”

In other instances, particularly in four agencies that ultimately did not succeed in implementing their EBP, the assessment of technical assistance and training was somewhat mixed at best. For instance, one agency director stated, “and I do think some of the staff through the training, um, didn't necessarily have the best experience with the trainer and I felt like there might have been some damage to their perception of…the effectiveness of the program because the trainer didn't necessarily do a good job getting buy in, um, you know getting the staff excited about a great program.” Furthermore, as explained by a clinical supervisor from the same agency, “you have 3 days where you're inundated with information, you're watching a lot of DVDs, you have the manual. But then it's like you leave and then it's like poof, go do it. So, for me, no. I don't learn that way. Because I don't feel like 3 days of training, onsite training and a manual, is ready…No. We didn't feel ready at all.”

A third important outer setting influence on the self-assessment of feasibility and desirability during the Implementation phase, was receipt of appropriate referrals. Some agencies began to solicit referrals soon after training was completed. One agency program director reported soliciting referrals 2 weeks after training: “We have a direct referral source from Probation, and since Juvenile Probation is the one who asked us to do the treatment they already had some families for our court.” According to the program director of one agency, “It has not always been 100% smooth, you know. We'd be wanting to have a case, we're ready for new cases 2 weeks from now, and it might take until 3 or 4 weeks before we get them. So, it's been a little bit, uh, challenging to maintain full client caseloads at all times.” For others, the period between training and referrals was much longer. The executive director of another agency reported that there were few referrals for the first 18 months after training and, “it was really slow. A lot of that was us having to go back to child welfare and having to educate them on what a good referral was. We had to spend a lot of time on that. And we still actually have to spend a lot of time on that.” Related was how to track and extend appropriate referrals. One agency elected to create a management system for tracking referrals, to “you know, [know] who goes where. Who are they seeing? Uhm, so I track that every week, and I update Probation on, you know, who has been referred, who are they working with, and that kind of stuff because Probation didn't actually have like a—an existing system for that. So, we created one.”

Decisions also were based on relationships with referral sources. For example, one agency described having to educate the child welfare agency in their county, “because part of the problem is that in our county, the way the referrals come in, through child welfare services, we rely on the child welfare staff to really have the reunification plan and then know about our programs and understand it, and then make the referrals. Otherwise we don't have any. And in the same token, to be able to educate our foster parents in terms of an option, actually create a stock of foster parents that are trained and ready in this way. Oh, that stakeholder meeting was very, very vital for us, and actually it still kind of is.” Referral barriers experienced by agencies during this phase of Implementation was another factor underlying these decisions. One agency noted “we had to get the type of licensure—one is family foster care, which anyone without any prior experience can apply for. Then there's the specialized license, which is required per our contract with the state. So I lose, probably I lose half right there…. Other barriers … some folks are maybe interested in chronic services, already have other children in the home. They might already be foster parents and they have other children in the home, which we cannot place if there's already a placement like that in the home…. To be honest some of the folks that maybe we thought were going to be a great fit, like current foster families, actually haven't been.”

Lack of community support was another barrier to ongoing Implementation. “We really did not want to end the service. It was more along the lines of we didn't have the community support to keep it going. So without that, I guess the mental health board supporting us, or someone else within the community speaking on our behalf that this really is the best service that we could provide… without someone coming forward to agree to help us do that, it was really hard to sell it…I think that's where we really got kind of stuck, was, how do we convince these people that this is the direction that everybody should go in?”

#### Inner setting

Influences associated with the inner setting were parallel to those identified in the self-assessment during Pre-Implementation and included the availability of existing staff or the need to hire new staff to administer the EBP with fidelity. As noted earlier, in some instances, agencies had existing staff who were trained to implement the EBP. As noted by one program director, “so, we've got two therapists that were already embedded into the agency doing other types of private and adult drug court work, and then they ended up, uh, they're just taking on…because the [EBP] is 6 months−4 to 6 clients every 6 months for us…” In other instances, additional staff were hired to implement the EBP. “The majority of our team was hired. So not only were we new to this agency, we were new to [EBP]. And we were all hired under the understanding that we would become certified [EBP] therapists.” In some instances, the need to hire additional staff was a consequence of high staff turnover. “I think our biggest internal problems have been turnover in our department.”

Similarly, the availability of experienced supervisors influenced Implementation progression. As explained by one agency program director, “Uh, but what was also very beneficial to us, and we didn't even really realize how beneficial it proved to be or would prove to be, [was] the supervisor that we hired for the program came to us from another [EBP] program…. She was new to the supervisory role in [EBP], but she had been doing [EBP] therapy for a couple of years. So, with the combination of her being available full-time for the new staff, the four new staff for whom [EBP] was brand-new, she was very experienced, and so that made it pretty seamless. Had we had somebody in a supervisory role who was also brand-new to the model, which I know many programs do, I think there would have been some different challenges… that would not have been as smooth.”

#### Interactions across setting

Finally, implementing the program with fidelity was a challenge encountered by the agencies during the Implementation phase that cross both the inner and outer settings. For some agencies, fidelity to the model did not fit with existing community standards for delivering services. As explained by one agency program director, “I think really what came from that is that the county was not willing to comply with the fidelity, for one. So the referrals that we were getting didn't quite fit in with the model and we were losing foster parents left and right because we were not providing them with placements.” For other agencies, lack of capacity to document fidelity was a major challenge. As the associate executive director of one agency observed, “Uploading the videos has been a challenge because our IT infrastructure, for whatever reason, cannot handle the capacity to upload them. So we've had some problems with that but I think we've finally resolved that. It required our IT director to get involved.”

### Sustainment phase (SIC stage 8)

The Sustainment Phase of the SIC does not include a full assessment of sustainability, but rather, if the agency is prepared to begin sustaining long-term (3). Assessment of the feasibility and desirability of sustaining the EBPs was influenced by a number of factors, including whether there were sufficient revenues to support the program, whether there was support from sources external to the agency, and whether there was a genuine need for the program. Further, whether the EBP was consistent with agency goals, and the costs and benefits of implementation impacted self-assessment of sustainment.

#### Intervention

Similar to Pre-Implementation, and Implementation phases, characteristics of the intervention influenced agency leader self-assessments of Sustainment. In many instances, agency leaders pointed to elements of EBPs that were not compensated. For instance, one program director stated, “That's something we're definitely working on…it's encouraged, you know, even though they might not do it after hours. They do text and call with their clients during the daytime, all the time. So there is a lot of hours outside of just sitting, doing therapy, that are involved with [EBP]. That, you know, there is at least some, not full, but some compensation for that…So, yeah, but recovering the costs of the extra hours, it's just a much more expensive type of therapy than your average.”

#### Inner setting

Inner setting considerations for the self-assessment of sustainment included whether or not the EBP was consistent with agency goals. As noted by one program director, “So we were already big believers of I guess [EBP] because we—we always treated the family. What this gives us is another option, another type of service to treat the family.” Moreover, the decision to sustain an EBP involved an assessment of the costs and benefits of implementation to the agency. One of the major costs identified by providers at this stage of implementation is the reduced revenue due to more intensive services delivered to fewer clients. According to one program director, “We get a lot of our revenue from group therapies, we run a ton of groups for our adult model… And so, you know, clinicians who are doing [EBP] don't run as many groups because they don't have the time or the capacity to do so because [EBP] takes up so much of their time. And so …, they have less of a case load of course and you know, don't run as many groups. So then that cuts into our revenue…”

On the other hand, agency leaders were able to identify benefits that could influence sustainment, “I think that it's given—it's opened our staff up to some new ideas and—and new ways of doing things, so I think that was definitely a benefit.” Similarly, another program director noted, “We've seen more benefits to it than—it's more positive for us than negative. It's a great system, it works really well with very specific families. We have really good outcomes. We're grateful that we had an opportunity to be trained in [EBP] and to use the model. The biggest barrier is the fact that sometimes we don't get reimbursed at the rate at which we would like to, and that it actually costs us to deliver the service.” Not surprisingly, this barrier carried more weight for agencies who were not achieving strong clinical outcomes, “The agency is committed to funding it, so the funding for the program I feel pretty secure about. But I don't know if in another year or 2 years if I don't see improvements in our outcomes and our outcomes matching more of what [EBP] research outcomes say, I can't say that I would be able to justify the ongoing cost…if I don't start seeing the outcomes in, you know, the next year to 18 months. I'm realistic in that I know we just started this, just completed the training and it is going to take the staff some time to really grow into the model. But I would like to see some improvements in our outcomes in the next year to 18 months or we are going to have to take a real serious look at is the cost, the cost of the program to justify the outcomes we are getting.”

#### Outer setting

Related to sufficient revenues to support the program is a consideration of the stability of that financial support from external sources. “The revenue source that, uh, is paying for the bulk of this here is something called…basically 60% of the revenue comes from federal and [state] dollars, and the other 40% then the state covers so, that we are… our cost recovery is a rate-based system. We worked with the consortium to set that rate. They in turn—we bill—they bill [the state]…you're getting about 55–60% of your costs, but because of the funding that we have here in [different state], we are able to recoup 100%. Part of it comes from the feds, the other part, the state kicks in so that we can be reimbursed fully.”

Another influence associated with the outer setting was whether there was a genuine need for the program. In all instances, participants expressed a need for evidence-based approaches to treatment in general and to the three participating EBPs in particular. “I saw that that was a big need. Our agency, with the youths that we work with… we had done individual and group therapy for years, with different evidence-based practices, but there was just a component that was missing, and it was definitely the family part. Uh, we'd had parenting classes but we needed much more than that.”

#### Interactions across settings

Not surprisingly, agency leaders' self-assessment of the potential to sustain the programs involved an interaction of both inner and outer setting characteristics. As noted by one program director, “I just reiterate that these are the higher level cases that typically if you didn't have something like this, and I would say, even go as far as to say if you didn't have this, then you would probably see kids fall through the cracks. They would go on to become, you know, at an adult level in the prison system, because these are kids that nobody else really, uh, knows how to help, or what to do with. And so, you know, it prevents that happening…them falling through the cracks. And it helps kids that, really, the outcomes usually are very bleak, have some better outcomes…So you are preventing all costs to the state in terms of the, you know, that detention cost for long term detention. And you are also preventing the youth going on to be adult, uh, involved with adult crimes.”

Finally, although fiscal barriers were noted across all three implementation phases, agency leaders were able to recognize that once the Sustainment phase was achieved, the impact of this barrier might be reduced through the interaction of inner and outer settings. “Wow, you know the initial training cost is the barrier. That's the biggest, the biggest cost. The ongoing costs, our costs for next year our continued certification is going to be like $8,000. Really in the scheme of things, that really isn't insurmountable at all…It really doesn't affect our revenue. The staffing costs are gonna be the same whether we do [EBP] or not, which is our biggest cost. Um, so anything additional… it really doesn't affect their productivity because their productivity with [EBP] and what we were asking from them before was not inconsistent.”

## Discussion

Qualitative interviews from this study supported the overarching premise that the SIC could accurately guide agency leaders in a self-assessment of the pre-implementation, implementation, and sustainment phases (Table 1). Responses from agency administrators indicated that activities identified on the SIC can accurately distinguish sites that proceeded with EBP implementation, and those that determined that progression was not appropriate for their agency. Extent of prior experience with implementing EBPs appeared to be a factor in implementation success: Four of the 15 agencies had no prior experience with using EBPs and three of these four agencies discontinued implementation of the selected EBP. Moreover, the SIC was able to identify implementation activities that, when asked about, highlight challenges and facilitators that contribute to the success, or not, of implementation efforts. Importantly, although the selected EBPs are similar in requirements and structure for program delivery (e.g., team approach, community-based, family treatment), it is illuminating that the SIC was able to provide a generalized framework that is applicable across interventions.

In this study, assessment of the feasibility and desirability of implementation of EBPs was found to involve different sets of influences: Those that occur during Pre-Implementation, Implementation, or at the beginning of Sustainment. All three phases revealed characteristics of the intervention, the inner and outer settings, and the interaction between settings. Pre-Implementation was influenced by intervention characteristics of affordability, feasibility, requirements, validity, reliability, relevance, cost savings, positive outcomes, and adequacy of information. Pre-Implementation outer setting characteristics included availability of funding, support from sources external to the agency, and adequacy of technical assistance. Inner setting characteristics of staff availability and attitudes toward innovation in general and EBPs in particular, organizational capacity, the fit between the EBP and agency mission and capacity, prior experience with implementation, experience with seeking evidence, and developing consensus. Self-assessments that occurred during the Implementation phase included intervention characteristics of flexibility and requirements, outer setting characteristics of availability of funding, adequacy of training and technical assistance, and getting sufficient and appropriate referrals; and inner setting characteristics of staffing and implementing with fidelity. During the Sustainment phase, assessments included intervention costs and benefits, outer setting characteristics of availability of funding, support from sources outside the agency, and need for the EBP; and the inner setting characteristic of the fit between the EBP and the agency mission.

The results offer four specific insights as to how agencies assess the feasibility and desirability of EBP implementation. First, consistent with the observation made by (5), different variables or influences might play crucial roles at different points of the implementation process. Availability of funding to support the EBP was a characteristic of the outer setting that influenced assessment of feasibility and desirability at all three implementation phases. EBP flexibility and requirements, adequacy of technical assistance from the treatment developer, and availability of qualified staff were important influences during the Pre-Implementation and Implementation phases but not the Sustainment phase. Assessment of costs and benefits of the EBP, support from sources external to the agency, need or demand for the EBP, and fit between the EBP and agency mission were influences on assessment of feasibility and desirability at the Pre-Implementation and Sustainment but not the Implementation phases. Also, the number of influences appeared to have grown smaller with each subsequent implementation phase (20 to 7 to 5), thereby suggesting that agency leaders weigh more considerations for continued implementation efforts during Pre-Implementation, than once the program has launched and is underway.

Second, the results highlight continuity of particular influences across all three implementation phases. Availability of funding was influential at all three phases. Assessment of whether there was sufficient information necessary to implement the EBP during the implementation phase was based on the information accessed and provided during Pre-Implementation. Assessment of the costs and benefits of the EBP conducted during the Sustainment phase was based on the experience during the Implementation phase.

Third, the results suggest that influences do not operate independently but in combination with one another. For instance, the inner setting degree of fit between different EBPs and the mission of the agency was an influence of assessment of feasibility and desirability that reflected an interaction between characteristics of the intervention (i.e., relevance) with the outer (i.e., demand for EBP) and inner settings. The availability of information about the EBP and its implementation was embedded in characteristics of the EBP itself as well as the outer (i.e., adequacy of technical assistance from treatment developer) and inner setting (experience with seeking evidence and information). The existence of such combinations suggests the need to examine potential mediation and moderation effects when identifying predictors of agency assessment of feasibility and desirability. For instance, evidence of EBP cost-effectiveness might impact level of support from sources outside the agency.

Finally, the assessments of EBP implementation feasibility and desirability are based on different forms of engagement, including engagement with other EBPs at the Pre-Implementation phase and engagement with information or evidence, and with other stakeholders at the Pre-Implementation and Implementation phases. An earlier study by Palinkas et al. (19) reported that personal experience was an important source of “evidence” used by systems leaders in deciding whether or not to implement EBPs. Clinical experience is one of the types of evidence used in the practice of evidence-based medicine (29). Use of research evidence was found in an earlier study to be significantly associated with the final stage achieved, as measured by the SIC, of TFCO by county-level youth-serving systems in California and Ohio (30). Engagement with other stakeholders both within the agency and external to the agency suggests that implementation is a “trans-relational” phenomenon involving interactions with other agencies (31, 32, 33), researchers (34, 35), and intermediaries (36).

### Limitations

This study has several limitations. As a qualitative investigation, the generalizability of these findings is limited to a sample of senior administrators of agencies serving children and adolescents. The specific needs and perspectives of this stakeholder group on assessment of EBP implementation feasibility and desirability will likely differ from those of other stakeholders. Surveys of a random sample of different stakeholder groups would increase the generalizability of these results. Further, the assessment was based on the implementation of three specific EBPs. It is unclear whether the findings could be generalized to other EBPs. Finally, we did not conduct follow-up interviews with study participants, thus limiting our ability to establish a causal linkage between assessment of feasibility and acceptability and potential influences.

## Conclusions

Despite the limited scope of this qualitative evaluation, our results support the conclusion that the relevance of implementation domains identified by most implementation models and frameworks vary by phase of implementation. Some of the influences on assessment of feasibility and desirability transcend more than one phase, while other influences appear to operate in combination with one another. Future research will consider if there is congruence between the quantitative data collected via the SIC, and the qualitative perspectives of agency leaders in their implementation process. Such evaluations will allow us to better assess and guide agencies toward informed decision-making and successful implementation.

## Ethics statement

This study was carried out in accordance with the recommendations of the Institutional Review Board at the Oregon Social Learning Center. The protocol was approved by the OSLC IRB. All subjects gave written informed consent in accordance with the Declaration of Helsinki.

## Author contributions

All authors contributed this article. LS is the Principal Investigator of the study. She conducted some of the qualitative interviews and engaged in manuscript preparation. MC is a research associate on the grant, conducted all of the qualitative interviews, transcription validation, and coding. LP is a co-investigator on the study and conducted the qualitative analyses, summarization, and manuscript preparation.

### Conflict of interest statement

The authors declare that the research was conducted in the absence of any commercial or financial relationships that could be construed as a potential conflict of interest.
